# Unveiling the probiotic potential of *L. rhamnosus* strain 044AE by genomic and phenotypic characterization

**DOI:** 10.3934/microbiol.2026007

**Published:** 2026-04-08

**Authors:** Aruna Inamdar, Vikash Kumar, Akanksha Chauhan, Yogini Dixit, Namrata Bhingardeve, Kunali Ambavale, Dina Saroj

**Affiliations:** Advanced Enzyme Technologies Limited, Sun Magnetica, Louiswadi, Thane-West, Maharashtra 400 604, India

**Keywords:** *L. rhamnosus* 044AE, probiotics, phenotypic assays, safety, whole genome sequencing

## Abstract

Probiotics are living microorganisms that improve overall human health by modulating the gut microbiota and enhancing absorption of nutrients and host immunity. Due to the growing demand for probiotics, efforts are being made to search for and characterize new probiotics. Whole genome sequencing has played a significant role in the identification and characterization of many probiotic candidates. In the present study, hybrid assembly (Illumina and Nanopore-based), genomic analyses, and phenotypic evaluations of *Lactobacillus rhamnosus* 044AE isolated from a dairy sample were carried out to investigate its probiotic potential. The assembled genome of *L. rhamnosus* 044AE showed maximum homology with *L. rhamnosus* BIO5326. Downstream analysis of the genome revealed safety, stability, and gut survival features. In the phenotypic assays, *L. rhamnosus* 044AE exhibited favorable adhesion, aggregation, gut stability (88.96% viability under fasting conditions; 92%–96% viability under fed conditions), antimicrobial, antioxidant, and enzyme activities. Therefore, *L. rhamnosus* 044AE appears as a potential probiotic candidate for applications in the food, pharmaceutical, and nutraceutical industries.

## 1. Introduction

Probiotics are usually live bacteria that exert beneficial effects on human health when consumed in appropriate amounts. Probiotics are advised to be taken during the treatment of microbial infections to maintain the body's natural microbiota, support food digestion, improve nutrient and vitamin absorption, and overcome food allergies. Moreover, probiotics are also utilized in the treatment of obesity [1], prevention of dental caries [2], reduction of cholesterol [3], and enhancement of host immunity [4].

Species belonging to two bacterial genera, *Bifidobacterium* and *Lactobacillus*, are widely used as probiotics [5]. Among the various probiotic *Lactobacillus* species, *L. rhamnosus* has been widely used and studied [6]. Although various *L. rhamnosus* strains (ATCC 53103, GG, LGG, GR-1, LB21, and 271) have been well-studied and some strains have been used in commercial products (like starter cultures in yogurt and dairy products), *L. rhamnosus* strain GG (Gorbach–Goldin) is one of the most well-documented probiotic microorganisms. It exhibits probiotic features such as resistance to acid and bile and good proliferation capacity, which allows it to thrive within the gastrointestinal environment [7],[8]. The present work aimed to characterize the *L. rhamnosus* strain 044AE to highlight its capabilities for robust industrial applications.

Whole genome sequencing (WGS) has emerged as a rapid and reliable technique for the identification and genotypic characterization of novel microorganisms [9],[10]. Regulatory agencies, such as EFSA, have made WGS mandatory for the safety analysis of microorganisms used as food [11]. WGS of potential probiotic strains not only facilitates correct taxonomic assignment but also provides information on the presence of genes responsible for probiotic traits, virulence factors, antibiotic resistance, and hazardous metabolites [12]–[14]. In the past, many potential probiotic strains have been identified and characterized by WGS [15],[16].

In this study, the genome of *L. rhamnosus* strain 044AE was characterized by both WGS-based genomic data and phenotypic assays to explore its probiotic potential. Analyses revealed that *L. rhamnosus* strain 044AE harbors multiple genes that contribute to its probiotic properties and lacks genes that could raise safety concerns.

## 2. Materials and methods

### 2.1. Genomic sequencing and assembly

*L. rhamnosus* strain 044AE was isolated from a dairy product and stored in glycerol vials at −80 °C. The quantity and quality of the genomic DNA obtained from *L. rhamnosus* strain 044AE were assessed using Nanodrop-2000/Qubit and agarose gel electrophoresis, respectively.

Whole genome sequencing of *L. rhamnosus* strain 044AE was carried out by both Illumina and Oxford Nanopore Technologies (ONT) (Figures S1–S3 and Tables S1–S4). The Unicycler assembler was used for de novo genome assembly [17] based on default parameters (-1 and -2 for Illumina paired-end reads, -l for nanopore reads, -t for number of threads, and -out for generating output). Unicycler v0.4.8 is a whole genome hybrid assembler tool that combines the efficiency of the de Bruijn graph and overlap–layout–consensus (OLC) approaches. The tool is designed to detect and correct single-base and short insertion/deletion errors in assembled genomes using Illumina data.

### 2.2. Genomic comparison

The assembled genome of *L. rhamnosus* strain 044AE was compared with other bacterial genomes present in the RefSeq genome database using NCBI-BLASTN [18]. The good-quality processed reads were mapped against the closest reference genome (*L. rhamnosus* strain BIO5326) using BWA -v0.7.17 to assess the mapping statistics.

Average nucleotide identity (ANI) analysis was carried out between the assembled genome of *L. rhamnosus* strain 044AE and the reference genome (*L. rhamnosus* strain BIO5326) using the online ANI calculator tool [19] and PYANI [20]. A synteny plot between the assembled genome of *L. rhamnosus* strain 044AE and the reference genome (*L. rhamnosus* strain BIO5326) was generated using MAUVE version 2.4.0 [21] based on default parameters. Mauve performs genome alignment to identify evolutionary changes in the DNA by aligning homologous sequence regions. Circular genome comparison between the two genomes was carried out using BRIG-V0.95 based on default parameters [22].

### 2.3. Gene prediction and annotation

The final assembled genome was used for all downstream analysis, which included gene prediction, GO annotation, pathway analysis, and identification of antibiotic-resistant genes, virulence factor genes, mobile elements, insert sequences, prophage sequences, biogenic amine-producing genes, and probiotic genes.

The assembled genome for *L. rhamnosus* 044AE was processed for gene prediction using the Prokka-V1.14 genome annotation [23] tool based on default parameters. Gene annotation was carried out for protein-coding genes; these annotated coding genes were processed for gene ontology against all the proteins from bacterial species (152,847,338) listed in UniProt (March 2022) (UniProt Consortium, 2017) database for bacteria. Pathway annotation of the predicted proteins was performed using the KAAS web server (KEGG Automatic Annotation Server, accession date: 23/09/2022) [24], which provides KEGG orthology information for all *Lacticaseibacillus rhamnosus* species listed in the database.

Comprehensive Antibiotic Resistance Database (CARD) [25] and Virulence Factor Database (VFDB) [26] were used to search antibiotic resistance genes and virulence factors, respectively. In both cases, BLASTX was used with the criteria (similarity > 80%, coverage > 70%) for the identification of significant hits. The assembled genome was also aligned to the COG database (accession date: 28/05/2022) [27] to identify gene function. Mobile element sequences were screened in the genome of *L. rhamnosus* strain 044AE using homology search against the mobile genetic elements (MGE) database [28]. PHASTER server [29] was used for prophage analysis (http://phast.wishartlab.com). The identification of decarboxylase genes was performed by maximum homology between the assembled genome and decarboxylase genes of *L. rhamnosus* 044AE from the UniProt database (accession date: 06/10/2022) (UniProt Consortium, 2017). The Conserved Domain Database (CDD) [30] was searched using the batch CD-option to identify genes that contribute to probiotic properties such as adhesion to gut mucosa, acid tolerance, bile salt tolerance, and environmental stress resistance.

### 2.4. Detection of biogenic amines and cytotoxicity activities

Detection of amino acid decarboxylase activity from *L. rhamnosus* strain 044AE was carried out on modified media [MRS media with glycerol (0.25%), bromocresol purple (0.006%), and arginine (0.1%)] as described by Chang et al. [31].

For cytotoxic activity analysis, 100 µL of Vero cells were treated with different volumes of cell-free supernatant of *L. rhamnosus* 044AE (10, 50, and 100 µL) in triplicate. Samples were incubated for 2 h with continuous shaking and then centrifuged at 800 × g for 10 min. The supernatant was carefully removed, and cells were suspended in 200 µL of extracellular bathing solution (EC buffer: phosphate-buffered saline, pH 7, containing 0.1% BSA and 0.01% sodium azide, supplemented with 1 µg/mL of RNAse and 5 µg/mL of propidium iodide). Fluorescence was measured in a microplate spectrofluorometer using excitation/emission wavelengths of 575/615 nm with a 9 nm slit for excitation and 15 nm for emission. Triton X-100 (0.1% final concentration) was added as the positive control, and cells without active agent were used as the negative control.

### 2.5. Acid, bile, and temperature stability (in vitro study)

The stability of *L. rhamnosus* 044AE cells was examined at various pH, bile concentrations, and temperatures. Assays are described in detail in the supporting information.

### 2.6. Cell surface properties and adhesion assays

Microbial adhesion to hydrocarbons (MATH) [32], auto-aggregation [33], co-aggregation [34], mucin adhesion [35], and adhesion to CaCO-2 cell line [36] assays were performed to investigate the ability to colonize and survive in the gastrointestinal tract. Assays for cell surface properties are described in detail in the supporting information.

### 2.7. Beta galactosidase activity and bile salt hydrolase activity (BSH)

*L. rhamnosus* 044AE cells were grown overnight, inoculated in sterile ONPG broth, and incubated at 37 °C for 24 h under aerobic conditions. Yellow coloration compared with uninoculated ONPG broth indicated β-galactosidase activity [37].

*L. rhamnosus* 044AE BSH activity was measured by cultivating it on soft MRS agar (pH 5.6, MRS broth 52.5 g/L, bacteriological agar 7.5 g/L oxoid, CaCl_2_ 0.37 g/L) (control). MRS soft agar is considered as BSH agar after supplementing with bile salts (0.3% w/v; Ox Bile, Himedia, India). For bile salt hydrolase activity, spot inoculation of 20 µL of overnight grown 044AE culture was done on BSH plates and MRS soft agar control plates, incubated at 37 °C for 72 h. Precipitation around the colony indicated BSH activity [38].

### 2.8. Antioxidant activity

The antioxidant activity by DPPH radical scavenging was evaluated using the 96-well microtiter plate technique reported by Cai et al. [39] with some modifications (see supporting information for detailed methodology).

### 2.9. Antimicrobial activity

Overnight culture of *L. rhamnosus* 044AE was combined with XAD16N beads and allowed to grow for five days at 37 °C on clarified MRS agar. Antimicrobial compound AMC adsorbed onto XAD16N beads was extracted using 80% isopropanol (IPA) containing 0.1% trifluoroacetic acid (TFA), concentrated 10 times by Rotavapor (Rotavapor® R-300, Buchi, Switzerland). The spot-on-the-lawn assay was used to examine *L. rhamnosus* 044AE ability to antagonize 16 pathogens, namely *Enterobacter cloacae* ATCC 13047, *Pasteurella multocida* ATCC 12945, *Escherichia coli* ATCC 9002 NCTC, *Escherichia coli* ATCC 700728, *Pseudomonas aeruginosa* ATCC 9027, *Salmonella enterica* ATCC 14028, *Bacillus cereus* ATCC 33019, *Clostridium sporogenes* NCIM-5125 (equivalent to ATCC 19404), *Salmonella* abony NCIM-2257 (equivalent to ATCC 6017 NCTC), *Klebsiella pneumoniae* ATCC BAA-1144, *Staphylococcus aureus* ATCC 6538P, *Listeria monocytogenes* ATCC 19115, *Bacillus circulans* ATCC 4516, *Bacillus subtilis* subsp. *spizizenii* ATCC 6633, *Clostridium difficile* ATCC 9689, and *Clostridium perfringens* ATCC® 13124™. The pathogenic cultures were procured from the respective culture collection centers. The obtained zone of inhibition (mm) against every pathogen was noted. Distilled water was taken as the negative control.

### 2.10. Stability of *L. rhamnosus* 044AE in static gut model conditions (in vitro study)

All experiments were conducted under aseptic conditions, using freshly generated digestive fluids (Tables S5–S8) [oral (SSF), gastric (SGF), and intestinal (SIF)], which included bile, enzyme solutions, and pH changes. The *L. rhamnosus* 044AE-supplemented diets were agitated at 50 rpm and 37 °C and exposed successively to simulated salivary fluid (2 min at pH 7.0), simulated stomach fluid (2 h at pH 3.0), and simulated intestinal fluid (2 h at pH 7.0). Following the specified gastrointestinal transit periods, 1.0 mL samples were taken out of the reaction flasks, and the pour plate method was used to evaluate viable activity [40].

### 2.11. Stability of liquid preparations of probiotic *L. rhamnosus* 044AE

Viable activity of *L. rhamnosus* 044AE was adjusted to 2 billion CFU/mL in matrices consisting of aqueous matrix (distilled water), sunflower oil (100%), MCT oil (Pharma grade-BASF-100%), and Simethicone (commercial product). These matrices are the most common ingredients of pediatric drops available in the market. Stability of probiotics with such matrices can help establish potential in pediatric product development. Samples were kept at 4 and 25 °C (relative humidity of 60%) for 180 days. Viable activity was analyzed at days 0, 30, 60, 90, 120, 150, and 180.

Viable activity of *L. rhamnosus* 044AE was expressed as log_10_ CFU/mL and was the average of three repeated measurements of three independent replicated experiments from three individual batch samples. Significant differences between activity means were calculated at p < 0.05 using two-way analysis of variance (ANOVA) followed by Dunnett multiple comparison using GraphPad Prism 10.6.1.

## 3. Results

### 3.1. *L. rhamnosus* 044AE showed maximum homology with *L. rhamnosus* strain BIO5326

**Figure 1.**
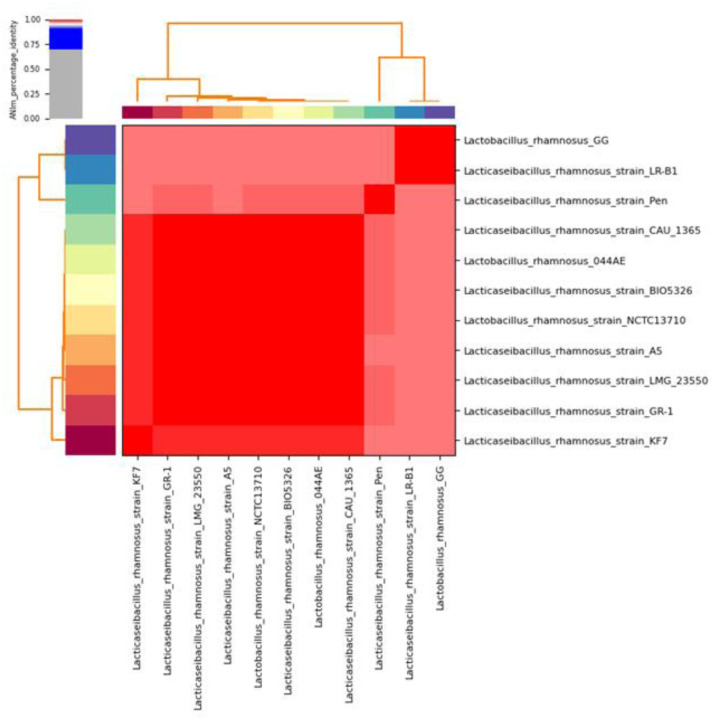
Heatmap showing the relationship between *L. rhamnosus 044AE* and other *L. rhamnosus* strains.

The de novo whole genome assembly for *L. rhamnosus* strain 044AE, using both Illumina and Nanopore reads, resulted in one contig of 2,990,904 bp in size with a GC content of 46.7%. The assembled genome was further compared with other bacterial genomes present in the RefSeq genome database using NCBI-BLASTN [18]. *L. rhamnosus strain* BIO5326 was chosen as the reference organism because BLASTN results indicated maximum homology with *L. rhamnosus* strain 044AE. 99.83% (4,978,535 reads) of the reads mapped against the above-mentioned bacterial reference genome, and 0.02% (8,478 reads) of the reads reported as unmapped for the specific reference bacterial genome. Genomic comparison of *L. rhamnosus* strain 044AE with other *L. rhamnosus* strains also suggested that *L. rhamnosus strain* BIO5326 is the closest strain (Figure 1).

**Figure 2.**
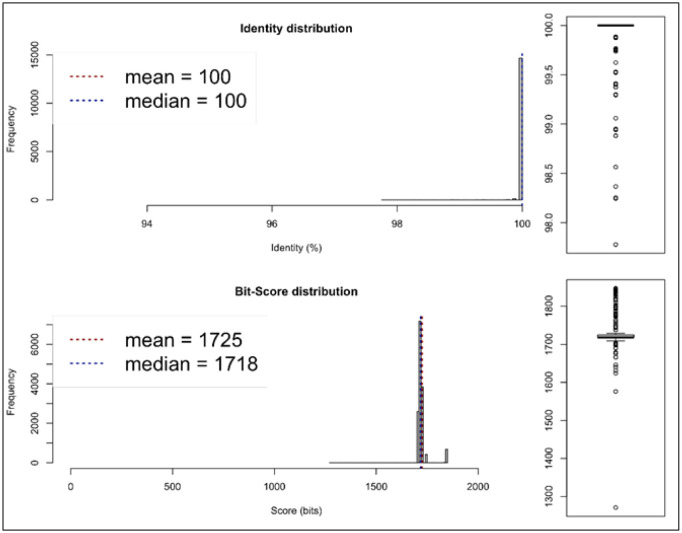
ANI plot comparison: *Lactobacillus rhamnosus* 044AE with *Lacticaseibacillus rhamnosus* BIO5326.

For all further homology plots, *L. rhamnosus* BIO5326 strain was used as the reference strain. It was observed that the overall sequence identity between the genomes was 100%. Average nucleotide identity (ANI) analysis was carried out between the assembled genome of *L. rhamnosus* strain 044AE and the reference genome (*L. rhamnosus strain* BIO5326) using the online ANI calculator tool [19], which indicated 100% identity between the two genomes. Another tool, i.e., pyANI, also gave an ANI value of 99.99%, close to 100%. The ANI output plot is shown in Figure 2. The circular comparison (Figure S4) shows similarity between *L. rhamnosus* strain 044AE and the reference (*L. rhamnosus* strain BIO5326) as concentric rings. BLAST pairwise alignment is used internally to check the alignment between genomes. The four rings represent GC content and GC skew for *L. rhamnosus* 044AE genome and the *L. rhamnosus* BIO5326 reference genome, as shown in Figure S4.

### 3.2. Genome annotation and cytotoxicity assay of *L. rhamnosus* 044AE revealed safety and probiotic features

**Figure 3.**
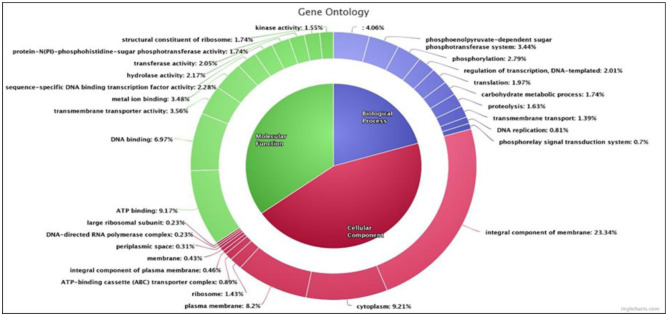
Gene ontology (GO) association of predicted protein coding genes for *L. rhamnosus* strain 044AE.

Gene prediction and annotation using Prokka deciphered a total of 2870 genes, which code for proteins, tmRNAs, tRNAs, miscRNAs, and rRNAs (Table S9). It was observed that, out of 2754 proteins from the assembled genome, 2746 had significant hits (>30% as identity and e-value as ≤1 × 10^−2^) against UniProt bacterial proteins. Gene ontology classification for these proteins indicated that 35%, 45%, and 20% of the proteins from the *L. rhamnosus* 044AE assembled genome could be assigned to molecular functions, cellular components, and biological processes, respectively (Figure 3). The abundance of proteins in these respective groups is indicated in Figures S5–S7.

It was observed that the most abundant protein was associated with a pathway related to transporters and ribosomes. Predicted proteins were associated with the pathway function groups viz. carbohydrate metabolism, membrane transport, amino acid metabolism, translation, replication and repair, and nucleotide metabolism, among others (Figure S8).

None of the antimicrobial and virulence factor genes satisfying the ≥80 identity and ≥70 coverage criteria were identified through the COG database. Table S10 lists a few important AMR and virulence genes that were absent in the *L. rhamnosus* 044AE. Moreover, the search against the mobile genetic elements (MGE) database did not show any hits, which leads to a potential risk of horizontal gene transfer. Genomic stability is an important safety indicator of probiotics [41]. The presence of prophage sequences in the bacterial genome makes them vulnerable to genomic changes. In *L. rhamnosus* 044AE, only two questionable phage regions were identified (Region1: 838398–881387 and Region2: 1048039–1062533). Mining of *L. rhamnosus* strain 044AE genome for safety-related genes revealed that the strain is safe.

Many proteins were identified through domain search, which may directly or indirectly govern the probiotic properties of *L. rhamnosus* 044AE. These have been categorized into mucosal adhesion, acid tolerance, bile tolerance, and environmental stress resistance (Table 1).

The *in vitro* cytotoxicity assay using Vero cell lines showed that the fluorescence values for the sample from *L. rhamnosus* 044AE were 20% lower than those of the positive control, indicating that the sample did not have any cytotoxic effect *in vitro* with 10–100 µL for a 2-h incubation period with Vero cells (Table 2).

### 3.3. Acid, bile, and thermal stability of *L. rhamnosus* 044AE (in vitro study)

Viable counts of *L. rhamnosus* cell suspensions (log_10_ CFU/mL) subjected to different acid concentrations are presented in Figure 4A. *L. rhamnosus* remains viable up to 4 h at pH 5.0 (P-value = 0.289) and up to 3 h at pH 3.0 (P-value = 0.297). At pH 2.5, viability is seen only up to 3 h (P-value = 0.064); at pH 1.5, there was a strict reduction in viability (Figure 4A).

Viable counts of *L. rhamnosus* cell suspensions (log_10_ CFU/mL) subjected to different bile concentrations are presented in Figure 4B. Viable activity of *L. rhamnosus* 044AE cells remained unaffected (initial viability 9.293 log_10_ CFU/mL) at bile concentrations of 0.01%–0.3% up to 2 h (P-value = 0.59). Viability at higher bile concentrations (0.5%–1%) was affected after 2 h of exposure. Viable counts of *L. rhamnosus* cell suspensions (log_10_ CFU/mL) subjected to different temperatures are presented in Figure 4C.

**Table 1.** List of proteins that contribute to the probiotic features of *L. rhamnosus* 044AE strain.

Probiotic feature	Identified domain using CDD
Adhesion to gut mucosa	MucBP (pfam06458)
	Sortase (pfam04203)
Acid tolerance	ATP synthase subunit a (pfam00119)
	ATP synthase subunit c (pfam00137)
	ATP synthase subunit alpha (pfam00006)
	ATP synthase subunit beta (pfam02874)
	ATP synthase subunit gamma (pfam00231)
	ATP synthase subunit delta (pfam00213)
	ATP synthase subunit epsilon (pfam02823)
	Orn/Arg decarboxylase (pfam01276)
Bile tolerance	Bile acid sodium symporter (pfam01758)
	Conjugated bile acid hydrolase (pfam02275)
Resistance to universal stress	Usp (pfam00582)
	Cpn60_TCP1 (pfam00118)
	Cpn10 (pfam00166)
	Hsp33 (pfam01430)
Resistance to heat stress	GrpE (pfam01025)
Resistance to hyperosmotic stress	PMSR (pfam01625)
Resistance to oxidative stress	
	DnaJ (pfam01556 and pfam0022))
	CLP_protease (pfam00574)
Resistance to cold shock	
	Csp (pfam00313)

**Table 2.** Fluorescence shown by *Lactobacillus rhamnosus* 044AE (cytotoxicity test).

Treatment	Fluorescence measurement	% fluorescence with respect to positive control
Positive control (0.1% Triton X-100)	71.941	100
Negative control	2.944	4.092
Background	2.437	3.388
10 µL (test sample)	2.877	3.999
50 µL (test sample)	2.848	3.958
100 µL (test sample)	3.237	4.499

**Figure 4.** Stability of *L. rhamnosus* (produced from *L. rhamnosus* 044AE) at different acidic pHs and bile concentrations for up to 5 h. The viable cell count is expressed in log_10_ CFU/mL ± SD (A, B). Thermal stability of *L. rhamnosus* (produced from *L. rhamnosus* 044AE) under different temperatures and times of incubation. The viable cell count is expressed in log_10_ CFU/mL ± SD (C).

*L. rhamnosus* 044AE preparation was stable in all tested experimental temperatures. Viability of *L. rhamnosus* 044AE cells remained unchanged between 4 and 50 °C up to 3 h (9.30 log_10_ CFU/mL, P-value = 0.71). A strain showing good tolerance to thermal stress or good stability under ambient storage conditions gets better industrial acceptance. Aqueous suspensions of *L. rhamnosus* 044AE showed stability under tested temperatures (4–50 °C), and the presence of proteins for cold and heat shock resistance (Table 1) further confirmed this property.

### 3.4. *L. rhamnosus* 044AE exhibits cell surface properties required for adhesion and aggregation

Microbial adhesion of *L. rhamnosus* 044AE to non-polar solvents is shown in Table S11. Auto-aggregation for *L. rhamnosus* 044AE was 7.34% (±0.94%) after 6 h. Study of cell surface properties revealed its significant co-aggregation with crucial pathogens like *C. perfringens* (45.4%) and *S. enterica* (16.38%) (Table S11).

*L. rhamnosus* 044AE adhered well to mucin *in vitro*. This method analyzes the ability to adhere to mucin by determining the significance between CFU/well adhered to agar control (1.92 ± 0.5 × 10^6^) and CFU/well adhered to agar + mucin (1.40 ± 0.7 × 10^7^). It is evident that there is a significant difference between the two means, indicating adhesion to mucin.

**Figure 5.**
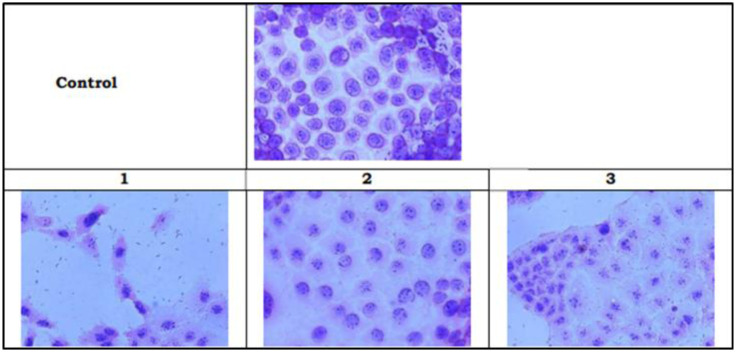
Adhesion of *L. rhamnosus* 044AE to Caco-2 cell culture as seen in three different fields (1, 2, and 3).

Cell adhesion potential of *L. rhamnosus* 044AE was evaluated on human epithelial cells (Caco-2 Cells). Cell adhesion potential was evaluated using two methods, i.e., observation of bacterial adhesion under the microscope (adhesion score) (Figure 5) and counting the adhered cell colonies after trypsinization (percent adhesion calculation). Based on observations of direct adhesion of bacteria to Caco-2 cells, the adhesion score of 044AE was 41.25 ± 7.11, indicating adhesion to human epithelial cells (Caco-2 cells). Percent adhesion data indicated a similar pattern, with 0.81% ± 0.05% adhesion to human epithelial cells (Caco-2 cells). Adhesion to Caco2 cells shows lower values, such as many potential probiotics, as this property is strain-specific, and the organism may use other mechanisms to persist in the GI tract.

### 3.5. Bile salt hydrolase and β-galactosidase activities of *L. rhamnosus* 044AE

**Figure 6.**
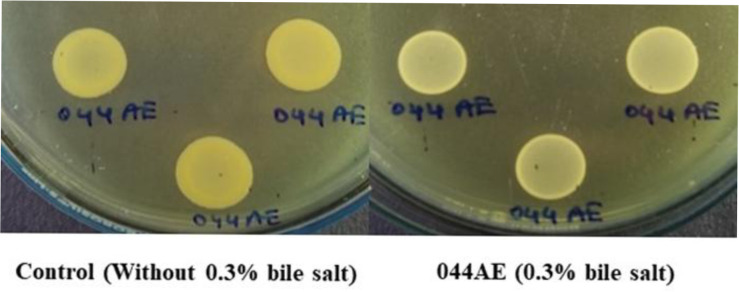
Bile salt hydrolase activity exhibited by *L. rhamnosus* 044AE.

Bile salt hydrolase of *L. rhamnosus* 044AE showed positive results, with a visible precipitate around the growth (Figure 6). *L. rhamnosus* 044AE also presented β-galactosidase activity (Figure S9).

### 3.6. Antioxidant assay by DPPH radical scavenging effect

DPPH free radical scavenging activity in *L. rhamnosus* 044AE (Table S12) supernatant indicates its additional functional property.

### 3.7. Antimicrobial activity

Antimicrobial activity was assessed against 16 pathogens by the spot-on-the-lawn assay method. 044AE AMC showed a zone of inhibition against 16 tested pathogens, and high (>15 mm) activity was seen against *Bacillus subtilis* subsp. *spizizenii* ATCC 6633, *Clostridium difficile* ATCC 9689, *Pasteurella multocida* ATCC 12945, *Pseudomonas aeruginosa* ATCC 9027, and *Clostridium perfringens* ATCC® 13124 (Table 3). The antimicrobial potential can be attributed to bioactive peptides that usually get extracted on XAD resin and eluted with IPA.

### 3.8. Stability of *L. rhamnosus* 044AE in static gut model conditions (in vitro study)

**Figure 7.**
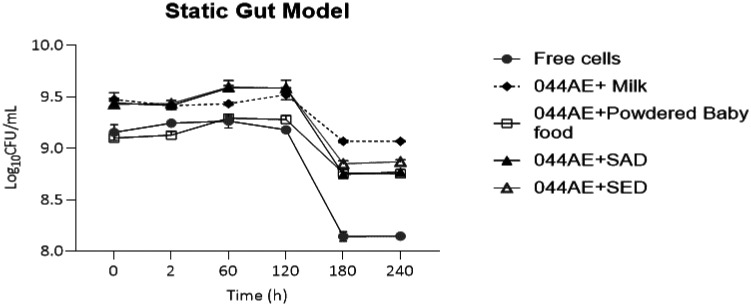
Orogastrointestinal stability of *L. rhamnosus* 044AE (log_10_ CFU/mL) as free cells and in the presence of food matrices through a static simulated gut model (*in vitro* study). SAD: Standard American diet; SED: standard European diet.

Free cells of *L. rhamnosus* 044AE were observed to be viable during oral, gastric, and intestinal phases of digestion (P-value ≤ 0.0029 for free cells at 180 min) (Figure 7). The overall viability at the end of the T digestion was 88.96%. The viability was unaffected in the salivary and gastric phases, and a slight reduction of 1.01 log_10_ CFU/mL in viability was observed during the intestinal digestion phase. The viability improved in the presence of food matrices and ranged between 92% and 96% with only a 0.3–0.5 log reduction. Thus, it can be inferred that *L. rhamnosus* 044AE is able to survive in simulated static gut model conditions under fed and fasting conditions.

**Table 3.** Zone of inhibition (mm) shown by *Lactobacillus rhamnosus* 044AE.

Sr. no.	Pathogen	044AE AMC mm	Positive control (20 µg/mL)
1.	*Enterobacter cloacae* ATCC 13047	11	36^CP^
2.	*Pasteurella multocida* ATCC 12945	16	34.5^CP^
3.	*Escherichia coli* ATCC 9002 NCTC	10	34^CP^
4.	*Escherichia coli* ATCC 700728	14	35.5^CP^
5.	*Pseudomonas aeruginosa* ATCC 9027	15	25.5^CP^
6.	*Salmonella enterica* ATCC 14028	14	34.5^CP^
7.	*Bacillus cereus* ATCC 33019	11	9^CP^
8.	*Clostridium sporogenes* NCIM-5125 (equivalent to ATCC 19404)	11	29^CP^
9.	*Salmonella abony* NCIM-2257 (equivalent to ATCC 6017 NCTC)	11	27.5^CP^
10.	*Klebsiella pneumoniae* ATCC BAA-1144	10	26^CE^
11.	*Staphylococcus aureus* ATCC 6538P	13	9^CP^
12.	*Listeria monocytogenes* ATCC 19115	11	15^CE^
13.	*Bacillus circulans* ATCC 4516	14	20.25^CP^
14.	*Bacillus subtilis* subsp. *spizizenii* ATCC 6633	19	25^CP^
15.	*Clostridium difficile* ATCC 9689	18	11.5^CP^
16.	*Clostridium perfringens* ATCC® 13124™	15	14.5^CP^

CP—Ciprofloxacin; CE—Cefixime; DW was taken as the negative control, which did not show any pathogen inhibition.

*L. rhamnosus* 044AE is able to survive in simulated static gut model conditions under fed and fasting conditions (in *in vitro* studies) and shows the presence of genetic domains coding for environmental stress-related proteins (Table 1), indicating its ability to adapt to the stressful conditions of different parts of the GI tract.

### 3.9. Stability of liquid preparations of probiotic *L. rhamnosus* 044AE

A stability study was conducted at two different temperatures (4 and 25 °C) using *L. rhamnosus* 044AE that was introduced into different liquid matrices (Figure S10). After 18 months, *L. rhamnosus* preparations under refrigeration had the maximum viability in MCT oil (96.25%), followed by sunflower oil (88.29%). *L. rhamnosus* 044AE can be stored under refrigeration in an aqueous matrix and Simethicone for 2 months, and MCT oil and sunflower oil for up to 18 months. *L. rhamnosus* preparations stored under accelerated storage conditions (25 °C, 60% RH) showed >50% viability for 8 months in sunflower oil (69.92% viability) and 10 months in MCT oil (67.73% viability).

## 4. Discussion

The study of the genetic makeup of bacterial strains aids in distinguishing probiotic strains from pathogenic strains. Genome-level characterization of a potential probiotic strain can provide important information about safety and genes related to probiotic properties. In the present study, an isolate from the dairy sample has been identified and named as *L. rhamnosus* 044AE after genotypic and phenotypic assessment. Both short reads and long reads were used to assemble the genome of *L. rhamnosus* 044AE. The assembled genome showed maximum homology with the genome of *L. rhamnosus* strain BIO5326 (Figures 1 and 2), and the GC content was similar. The ANI calculator considers only the aligned regions of DNA to calculate the average identity across genomes. A value of 100% indicates that *L. rhamnosus* 044AE is very close to the *L. rhamnosus* BIO5326. *L. rhamnosus* strain BIO5326 has been previously reported to decrease the epithelial to mesenchymal transition induced by *Helicobacter pylori* infection [42].

Genome annotation of *L. rhamnosus* 044AE did not reveal any antibiotic resistance gene with the risk of horizontal gene transfer. Lack of virulence factor genes further substantiated the safety of the strain. In a similar study by Zhao et al., *L. rhamnosus* x253 was observed to be devoid of virulence factor genes [43].

The ability of probiotics to survive under high bile salt concentrations and low pH is an important feature for their successful passage through the gastrointestinal tract. *L. rhamnosus* 044AE cells showed good stability in an acidic environment at pH 2.5 and very good tolerance to bile concentrations up to as high as 1.0%; the proteins responsible for these traits were identified (Table 1). In a previous study, the role of ATPase activity in acid tolerance was reported [44]. Similar results were reported by Rajoka et al. [45], who observed that the acid survival rates of all isolated strains of *L. rhamnosus* were higher at pH 3.0 than at pH 2.0. *L. rhamnosus* and *L. paracasei* isolates showed tolerance to different levels of bile salt (0.3%, 0.5%, and 1%) after 3 h of incubation. The thermal resistance of various other *L. rhamnosus* strains has been studied, and the outcomes are similar to *L. rhamnosus* 044AE. According to a previous study, *Lactobacillus* strains showed the ability to adapt and grow in a wide spectrum of temperatures, resulting in enhanced growth of *L. plantarum* LOCK 0860 and *L. pentosus* LOCK 1094 at 30 °C [46]. The presence of cold-shock and heat-shock protein genes, such as *grpE* and *csp* (Table 1), could be responsible for *L. rhamnosus* 044AE's ability to withstand thermal stress. *L. rhamnosus* 044AE revealed production of bile salt hydrolase, indicating an ability to hydrolyze bile salts, which is indicative of its potential role in cholesterol reduction. Probiotic bacteria able to produce β-galactosidase are presumed to have a role in alleviating symptoms of lactose intolerance. Similar to *L. rhamnosus* 044AE, *L. rhamnosus* GG has been reported to produce β-galactosidase [35],[47].

*L. rhamnosus* 044AE showed auto-aggregation (accumulation and precipitation of cells to aid the prevention of their quick removal due to intestinal peristaltic movements), co-aggregation with crucial pathogens, and adhesion to non-polar solvents. Adhesion of *L. rhamnosus* 044AE to mucin and Caco-2 cell lines (in *in vitro* tests) and the presence of genes that facilitate adhesion to gut mucosa (Table 1) further indicate its efficient colonization and survival in the GI tract. This is consistent with studies reported by Dias & Palaniswamy [48]. According to Rajoka et al., two isolates of *L. rhamnosus* showed high affinity to xylene, auto-aggregation, and co-aggregation abilities with *E. coli* and *Salmonella*, indicating suitability for human use [45].

There are reports of antagonistic effects of *Lactobacillus* strains against *E. coli*, *S. aureus*, and *Salmonella typhimurium* [37],[38],[49]. Probiotics can directly inhibit growth or kill pathogens by production of antimicrobial molecules, including short-chain fatty acids (SCFA), bacteriocins, or microcins, or by simply lowering the luminal pH [50]. Further purification and characterization studies would be needed to identify the antimicrobial compound produced by *L. rhamnosus* 044AE.

Similar to *L. rhamnosus* 044AE, the evaluation of DPPH activity of *Lactobacillus fermentum* has been done by Palaniswamy et al. [40]. High DPPH (2,2-diphenyl-1-picrylhydrazyl) radical scavenging activity in probiotics indicates their ability to neutralize free radicals and reduce oxidative stress in the gut. This antioxidant effect can help protect intestinal cells, support gut barrier integrity, and lower inflammation.

The survival profiles of six probiotic *L. rhamnosus* strains in the upper GI tract using an *in vitro* gastric model of digestion have been reported by Pitino et al. [33].

Probiotics that are stable in liquid formulations can be administered to infants or employed in the food and beverage sector. *L. rhamnosus* 044AE showed good stability in liquid matrices like MCT oil and sunflower oil. These findings show that it could be an ideal candidate for a new tailored therapeutic approach.

In the present work, *L. rhamnosus* 044AE showed excellent safety and stability features in whole genome sequence analysis and compatible probiotic functionalities like good GI persistence and survivability, as well as stability over a temperature range and in commercially important liquid matrices when studied *in vitro*. This indicates its potential for human and animal applications. Further *in vivo* validations are needed to create a strong ground for such food, feed, and pharmaceutical applications.

## Use of AI tools declaration

The authors declare they have not used Artificial Intelligence (AI) tools in the creation of this article.



## References

[1] Kobyliak N, Conte C, Cammarota G (2016). Probiotics in prevention and treatment of obesity: A critical view. Nutr Metab (London).

[2] Lopes PC, Gomes ATPC, Mendes K (2024). Unlocking the potential of probiotic administration in caries management: A systematic review. BMC Oral Health.

[3] Han MM, Sun JF, Su XH (2019). Probiotics improve glucose and lipid metabolism in pregnant women: A meta-analysis. Ann Transl Med.

[4] Mazziotta C, Tognon M, Martini F (2023). Probiotics mechanism of action on immune cells and beneficial effects on human health. Cells.

[5] Latif A, Shehzad A, Niazi S (2023). Probiotics: Mechanism of action, health benefits and their application in food industries. Front Microbiol.

[6] Mathipa-Mdakane MG, Thantsha MS (2022). *Lacticaseibacillus rhamnosus*: A suitable candidate for the construction of novel bioengineered probiotic strains for targeted pathogen control. Foods.

[7] Apizadeh M, Nahrevanian H, Rohani M (2016). *Lactobacillus rhamnosus* Gorbach-Goldin (GG): A top well-researched probiotic strain. J Med Bacteriol.

[8] de Champs C, Maroncle N, Balestrino D (2003). Persistence of colonization of intestinal mucosa by a probiotic strain, *Lactobacillus casei* subsp. *rhamnosus* Lcr35, after oral consumption. J Clin Microbiol.

[9] Hasman H, Saputra D, Sicheritz-Ponten T (2014). Rapid whole-genome sequencing for detection and characterization of microorganisms directly from clinical samples. J Clinl Microbiol.

[10] Peterson SW, Demczuk W, Martin I (2023). Identification of bacterial and fungal pathogens directly from clinical blood cultures using whole genome sequencing. Genomics.

[11] European Food Safety Authority (EFSA) (2024). EFSA statement on the requirements for whole genome sequence analysis of microorganisms intentionally used in the food chain. EFSA J.

[12] Guinane CM, Crispie F, Cotter PD (2016). Value of microbial genome sequencing for probiotic strain identification and characterization: Promises and pitfalls. The Gut-Brain Axis.

[13] Kim E, Yang SM, Kim D (2022). Complete genome sequencing and comparative genomics of three potential probiotic strains, *Lacticaseibacillus casei* FBL6, *Lacticaseibacillus chiayiensis* FBL7, and *Lacticaseibacillus zeae* FBL8. Front Microbiol.

[14] Li B, Da J, Smith EE (2017). Safety assessment of *Lactobacillus helveticus* KLDS1. 8701 based on whole genome sequencing and oral toxicity studies. Toxins.

[15] Saroj DB, Ahire JJ, Shukla R (2023). Genetic and phenotypic assessments for the safety of probiotic *Bacillus clausii* 088AE. 3 Biotech.

[16] Saroj DB, Upadhyaya P, Kumar V (2024). Whole genome sequencing-based genetic characterization and safety assessment of probiotic *Bacillus subtilis* strain PLSSC. Adv Biochem Biotechnol.

[17] Wick RR, Judd LM, Gorrie CL (2017). Unicycler: Resolving bacterial genome assemblies from short and long sequencing reads. PLoS Comput Biol.

[18] Altschul SF, Gish W, Miller W (1990). Basic local alignment search tool. J Mol Biol.

[19] Goris J, Konstantinidis KT, Klappenbach JA (2007). DNA-DNA hybridization values and their relationship to whole-genome sequence similarities. Int J Syst Evol Microbiol.

[20] Pritchard L, Glover RH, Humphris S (2016). Genomics and taxonomy in diagnostics for food security: Soft-rotting enterobacterial plant pathogens. Anal Methods.

[21] Darling AC, Mau B, Blattner FR (2004). Mauve: Multiple alignment of conserved genomic sequence with rearrangements. Genome Res.

[22] Alikhan NF, Petty NK, Ben Zakour NL (2011). BLAST Ring Image Generator (BRIG): Simple prokaryote genome comparisons. BMC Genomics.

[23] Seemann T (2014). Prokka: Rapid prokaryotic genome annotation. Bioinformatics.

[24] Moriya Y, Itoh M, Okuda S (2007). KAAS: An automatic genome annotation and pathway reconstruction server. Nucleic Acids Res.

[25] Jia B, Raphenya AR, Alcock B (2017). CARD: Expansion and model-centric curation of the comprehensive antibiotic resistance database. Nucleic Acids Res.

[26] Chen L, Yang J, Yu J (2004). VFDB: A reference database for bacterial virulence factors. Nucleic Acids Res.

[27] Tatusov RL, Galperin MY, Natale DA (2000). The COG database: A tool for genome-scale analysis of protein functions and evolution. Nucleic Acids Res.

[28] Pärnänen K, Karkman A, Hultman J (2018). Maternal gut and breast milk microbiota affect infant gut antibiotic resistome and mobile genetic elements. Nat Commun.

[29] Zhou Y, Yongjie L, Karlene HL (2011). PHAST: A fast phage search tool. Nucleic Acids Res.

[30] Marchler-Bauer, Bryant SH (2004). CD-Search: Protein domain annotations on the fly. Nucleic Acids Res.

[31] Chang M, Chang HC (2012). Development of a screening method for biogenic amine producing *Bacillus* spp. Int J Food Microbiol.

[32] Rosenberg M, Gutnick D, Rosenberg E (1980). Adherence of bacteria to hydrocarbons: A simple method for measuring cell-surface hydrophobicity. FEMS Microbiol Lett.

[33] Pitino I, Randazzo CL, Cross KL (2010). Survival of *Lactobacillus rhamnosus* strains in the upper gastrointestinal tract. Food Microbiol.

[34] Caggia C, De Angelis M, Pitino I (2015). Probiotic features of *Lactobacillus strains* isolated from Ragusano and Pecorino Siciliano cheeses. Food microbiol.

[35] Vinderola CG, Reinheimer JA (2003). Lactic acid starter and probiotic bacteria: A comparative ‘*in vitro*’ study of probiotic characteristics and biological barrier resistance. Food Res Int.

[36] Jacobsen CN, Rosenfeldt Nielsen V, Hayford AE (1999). Screening of probiotic activities of forty-seven strains of *Lactobacillus* spp. by *in vitro* techniques and evaluation of the colonization ability of five selected strains in humans. Appl Environ Microbiol.

[37] Fayol-Messaoudi D, Berger CN, Coconnier-Polter MH (2005). pH- Lactic acid- and non-lactic acid-dependent activities of probiotic Lactobacilli against *Salmonella enterica* Serovar *Typhimurium*. Appl Environ Microbiol.

[38] Liévin V, Peiffer I, Hudault S (2000). Bifidobacterium strains from resident infant human gastrointestinal microflora exert antimicrobial activity. Gut.

[39] Cai J, Bai J, Luo B (2022). *In vitro* evaluation of probiotic properties and antioxidant activities of Bifidobacterium strains from infant feces in the Uyghur population of northwestern China. Ann Microbiol.

[40] Palaniswamy SK, Vijayalakshmi G (2016). *In-vitro* probiotic characteristics assessment of feruloyl esterase and glutamate decarboxylase producing *Lactobacillus* spp. isolated from traditional fermented millet porridge (kambu koozh). LWT-Food Sci Technol.

[41] Wang Y, Liang Q, Lu B (2021). Whole-genome analysis of probiotic product isolates reveals the presence of genes related to antimicrobial resistance, virulence factors, and toxic metabolites, posing potential health risks. BMC Genomics.

[42] Jauvain M, Lepied G, Bénéjat L (2024). Effect of *Lactobacillus gasseri* BIO6369 and *Lacticaseibacillus rhamnosus* BIO5326 on gastric carcinogenesis induced by *Helicobacter pylori* infection. Helicobacter.

[43] Zhao L, Zhang Y, Liu Y (2023). Assessing the safety and probiotic characteristics of *Lacticaseibacillus rhamnosus* X253 via complete genome and phenotype analysis. Microorganisms.

[44] Bang M, Oh S, Lim KS (2014). The involvement of ATPase activity in the acid tolerance of *Lactobacillus rhamnosus* strain GG. Int J Dairy Technol.

[45] Rajoka MS, Mehwish HM, Siddiq M (2017). Identification, characterization, and probiotic potential of *Lactobacillus rhamnosus* isolated from human milk. LWT-Food Sci Technol.

[46] Śliżewska K, Chlebicz-Wójcik A (2020). Growth kinetics of probiotic *Lactobacillus* strains in the alternative, cost-efficient semi-solid fermentation medium. Biology.

[47] Li E, Zhu Q, Pang D (2022). Analysis of *Lactobacillus rhamnosus* GG in mulberry galacto-oligosaccharide medium by comparative transcriptomics and metabolomics. Front Nutr.

[48] Dias F, Duarte W, Schwan R (2013). Evaluation of adhesive properties of presumptive probiotic *Lactobacillus plantarum* strains. Biosci J.

[49] Nurhajati T, Soepranianondo K, Lokapirnasari WP (2017). Identification and characterization indigenous of *Lactobacillus* sp from bovine rumen fluid of slaughterhouse. KnE Life Sci.

[50] Ohland CL, MacNaughton WK (2010). Probiotic bacteria and intestinal epithelial barrier function. Am J Physiol-Gastr L.

