# Supplementary material for: Unveiling the probiotic potential of L. rhamnosus strain 044AE by genomic and phenotypic characterization
Source: AIMS Microbiol. 2026 Apr 8;12(2):173–91. doi: 10.3934/microbiol.2026007 (PMC13370279; doi:10.3934/microbiol.2026007)
Supplement: Supplementary file 1 [file microbiol-12-02-007-s001.pdf]

AIMS Microbiology

AIMS Microbiology, 12(2): 173–191.
DOI: 10.3934/microbiol.2026007
Received: 05 December 2025
Revised: 16 March 2026
Accepted: 25 March 2026
Published: 08 April 2026

https://www.aimspress.com/journal/microbiology

Research article

Unveiling the probiotic potential of *L. rhamnosus* strain 044AE by genomic and phenotypic characterization

Aruna Inamdar*, Vikash Kumar, Akanksha Chauhan, Yogini Dixit, Namrata Bhingardeve, Kunali Ambavale and Dina Saroj

Advanced Enzyme Technologies Limited, Sun Magnetica, Louiswadi, Thane-West, Maharashtra 400 604, India

* **Correspondence:** E-mail: aruna.inamdar@advancedenzymes.com; Tel: +91227506161194, 9004641267.

Supplementary

1. Library preparation for short read (Illumina) sequencing

Illumina library preparation was performed using QIASeq FX DNA Library Preparation protocol (Cat#180475) (Figure S1). Briefly, 50 ng of Qubit quantified DNA was enzymatically fragmented using the nuclease-enzyme cocktail supplied in-line with the library preparation kit. The fragmentation protocol entailed incubation of the template DNA with the enzyme mix at 32 °C for 16 min (this yields fragmented DNA of size in the range 200–300 bp) followed by 30 min incubation at 65 °C (and infinite hold at 4 °C). Fragmented DNA was end-repaired and A-tailed in a one-tube reaction using the FX Enzyme/ligation Mix provided in the QIASeq FX DNA kit. The end-repaired and adenylated fragments were subjected to adapter ligation, whereby index-incorporated Illumina adapter was ligated, to generate sequencing library. This library was subjected to 6 cycles of Indexing-PCR (Initial Denaturation at 98 °C for 20 sec, cycling (98 °C for 20 sec, 60 °C for 30 sec, 72 °C for 30 sec) and final extension at 72 °C for 1 min) to enrich the adapter-tagged fragments. Finally, the amplified library was purified using JetSeq Magnetic Beads (Bio, # 68031).

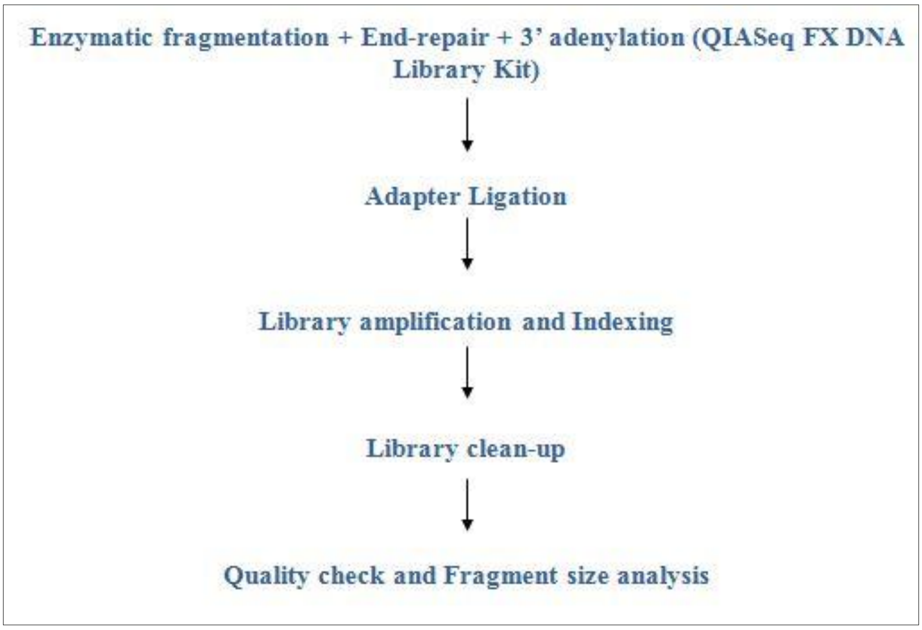

Figure S1. QIASeq FX DNA Library Preparation Protocol (HB-2015-002_1102703, May 2016).

The Illumina-compatible sequencing library was quantified by Qubit fluorometer (Thermo Fisher Scientific, MA, USA) and the fragment size distribution was analysed on Agilent TapeStation (Figure S2).

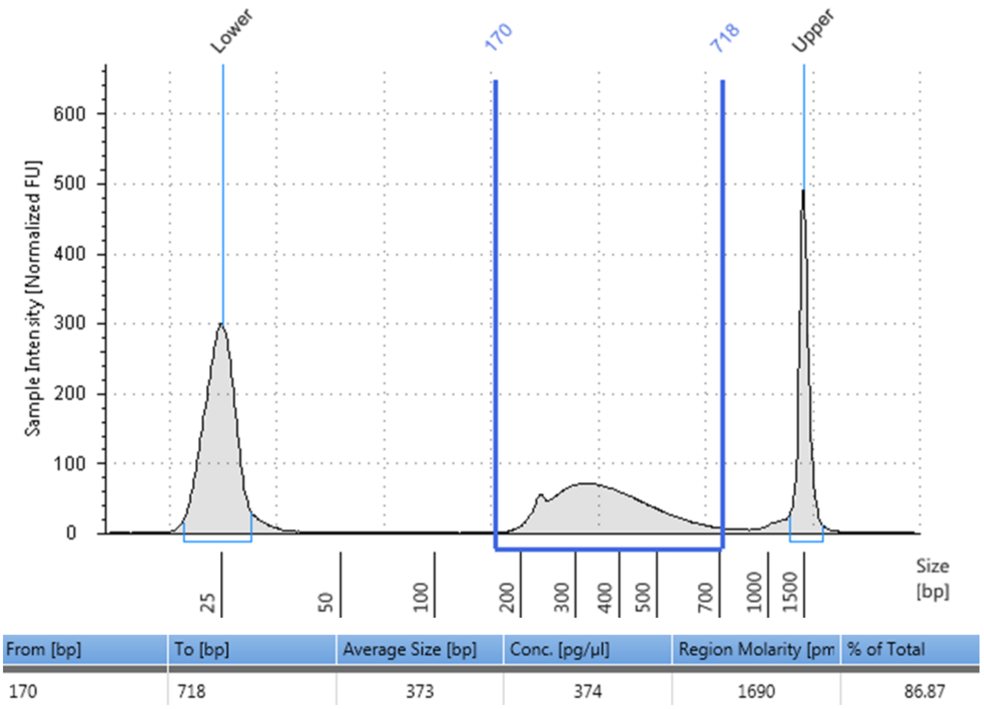

Figure S2. Tape Station profile of the Illumina library of *L. rhamnosus* 044AE.

The Illumina-compatible sequencing library showed average fragment sizes ranging between 170 bp and 718 bp, with enrichment around 373 bp. Given a combined adaptor size of approximately 120 bp, the effective user-defined insert size was estimated for the sample to be in the range 80 bp and 880 bp. The library thus constructed showed optimal concentration and qualified as suitable for Illumina sequencing to get the desired amount of sequencing data. Further, the library was molar-normalised and pooled to enable multiplex sequencing. Table S1 outlines the index used for barcoding the library.

Table S1. Barcodes used for Illumina sequencing

Sl.No.	Sample ID	Qubit Conc. (ng/uL)	Vol (uL)	Yield (ng)	Barcode 1	Index Sequence 1	Barcode 2	Index Sequence 2
1	*L. rhamnosus* 044AE	19.7	10	197	D712	AGCGATAG	D508	GTACTGAC

2. Library preparation for long read (Nanopore) sequencing

A total of 600 ng of purified total DNA from the sample was end-repaired (NEBNext Ultra II end repair kit, New England Biolabs, MA, USA); cleaned up with 1x AMPure beads (Beckmann Coulter, USA). Native barcode ligation was performed with NEB blunt/TA ligase (New England Biolabs, MA, USA) using EXP-NBD114 (ONT) and cleaned with 1xAmPure beads. Barcode sequence is detailed in Table S2.

Table S2. Barcode used for Nanopore sequencing.

Sample ID	Barcode name	Sequence
L. rhamnosus 044AE	NB40	TAGTTTGGATGACCAAGGATAGCC

Qubit quantified barcode ligated DNA sample was Adapter ligated for 15 minutes using NEBnext Quick Ligation Module (New England Biolabs, MA, USA). The library was cleaned up using 0.6X AmPure beads (Beckmann Coulter, USA), eluted in 15 µL of elution buffer and used for sequencing. Figure S3 illustrates the overview of native barcode library preparation.

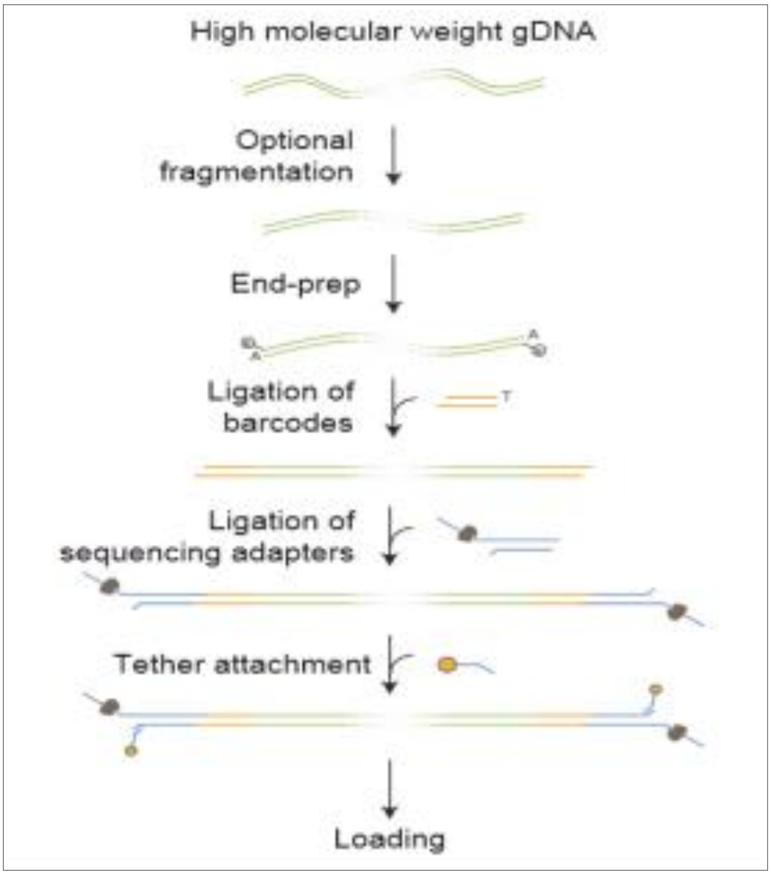

Figure S3. overview of native barcoding library preparation.

3. Sequencing for long read using Nanopore

Sequencing was performed on GridION X5 (Oxford Nanopore Technologies, Oxford, UK) using SpotONflow cell (R9.4) in about 15hrs using sequencing protocol on MinKNOW 2.1 v18.08.3 with default parameters (quality score > 7, Output file format selected: Fast5 and fastq fast5 bin size: 4000 files, fastq file bin size 4000 files). Nanopore raw reads (‘fast5’ format) were basecalled (‘fastq’ format) and demultiplexed using Guppy v2.3.4 with default parameters (Minimum phred score of 7, path for fast5 files, number of threads). After base calling, reads with a phred score (Q-Score) greater than 7 were retained as high-quality reads which were used for downstream analysis.

Table S3. Nanopore read statistics.

Data type	Rawdata	Processed
Reads Generated	452380	366807
Maximum Read Length	63321	60056
Minimum Read Length	102	9
Average Read Length	2277.7	2180.9
Median Read Length	12516	2168
Total Reads Length	1030397966	799961376
Total Number of Non-ATGC Characters	0	0
Percentage of Non-ATGC Characters	0	0
Reads >= 100 bp	452380	366291
Reads >= 200 bp	451602	352436
Reads >= 500 bp	385053	297091
Reads >= 1 Kbp	285631	219682
Reads >= 10 Kbp	11399	8863
Sequencing Coverage (X)	343	267

4. Sequencing for short reads using Illumina technology

Around 2.82 million reads were generated from Illumina NovaSeq platform with 501X coverage for sample *L. rhamnosus* 044AE (Table S4). Raw data was pre-processed using Trim Galore version 0.4.0 with different parameters (Minimum phred score of 30, minimum read length of 20, default universal adapter removal, number of base pairs before trimming is 150 and after trimming is between 20 to 150) and quality was calculated using FastQC (version 0.11.9). Phred quality score of pre-processed data was Q30.

Table S4. Illumina read statistics.

Sample Name	AETL63
Raw Reads	5013434 (5.0 Mb)
Pre-processed	4987013 (4.9 Mb)
Sequencing Coverage (X)	501

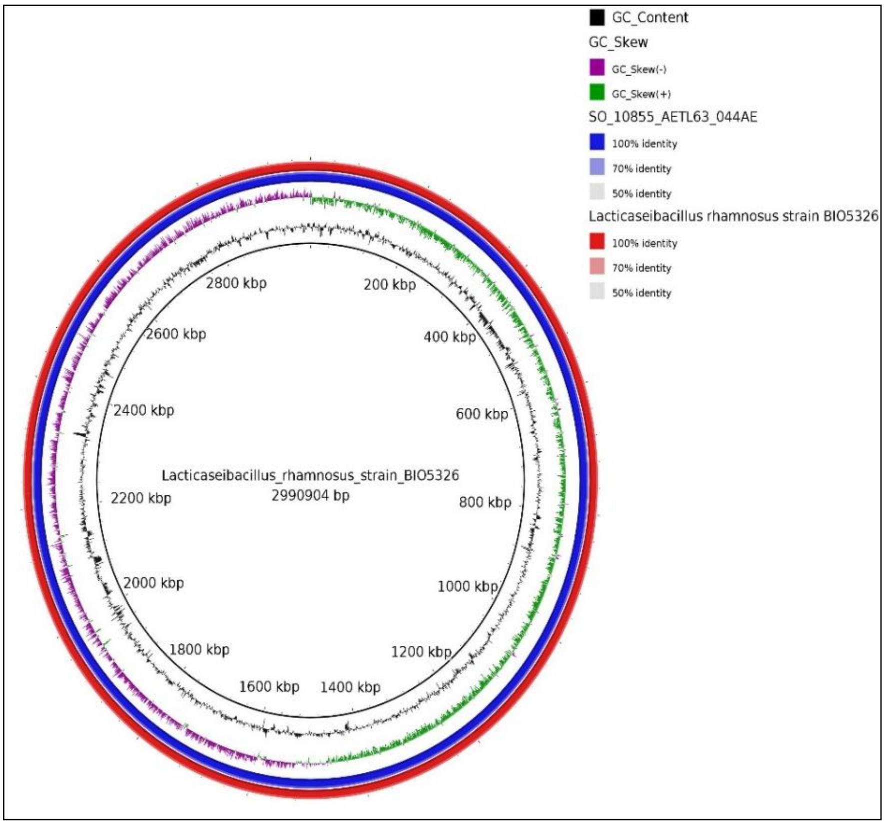

Figure S4. Circos plot comparison of *L. rhamnosus* strain 044AE (size of genome is 4204670 bp) with *L. rhamnosus* strain BIO5326.

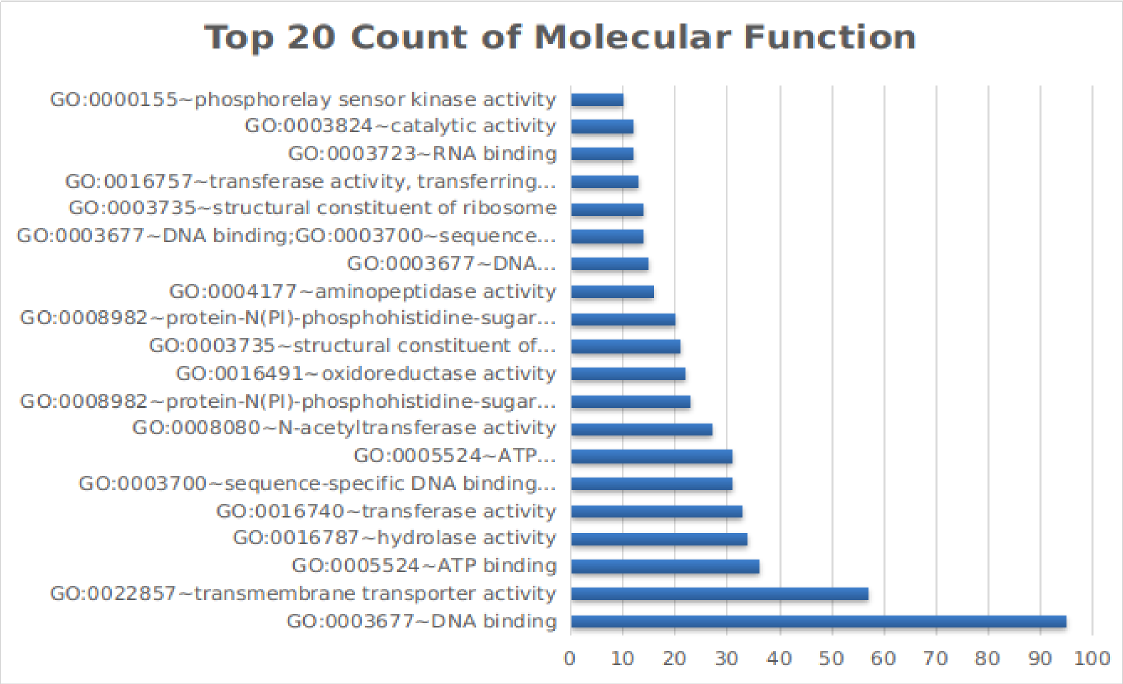

Figure S5. Top 20 Gene Ontology (GO) functions involved in molecular function for *L. rhamnosus* 044AE.

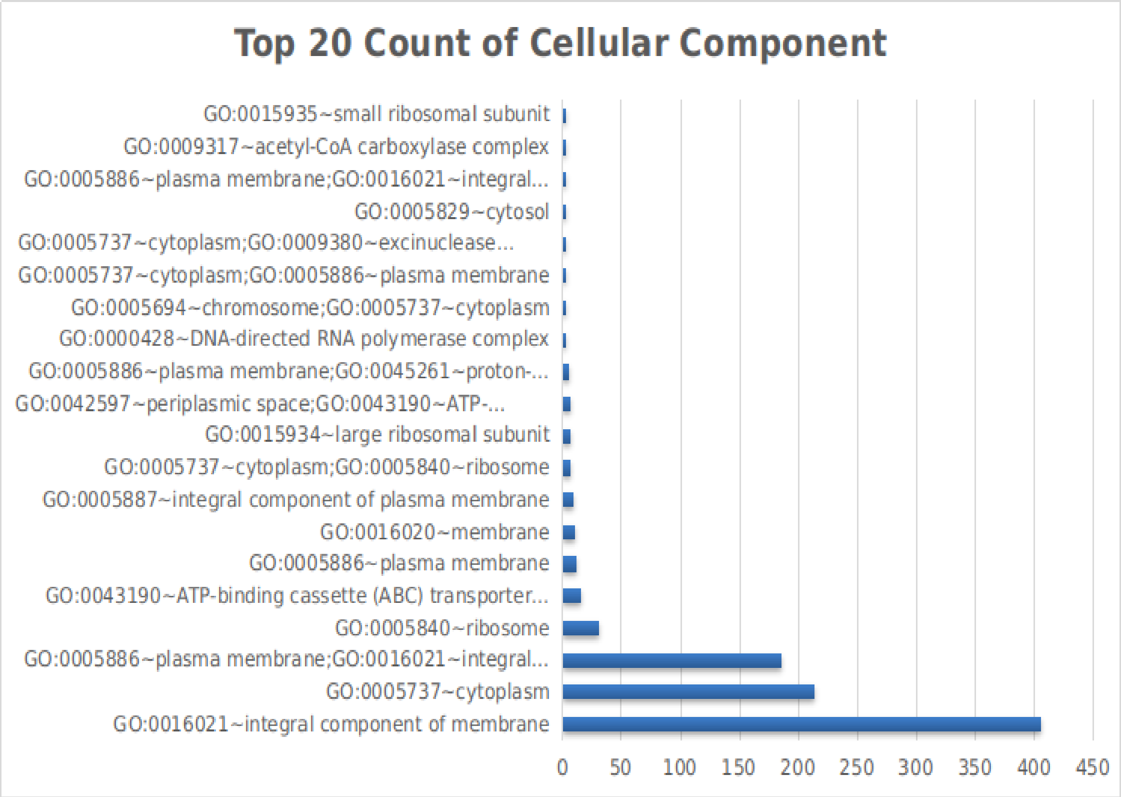

Figure S6. Top 20 Gene Ontology (GO) functions involved in cellular component for *L. rhamnosus* 044AE.

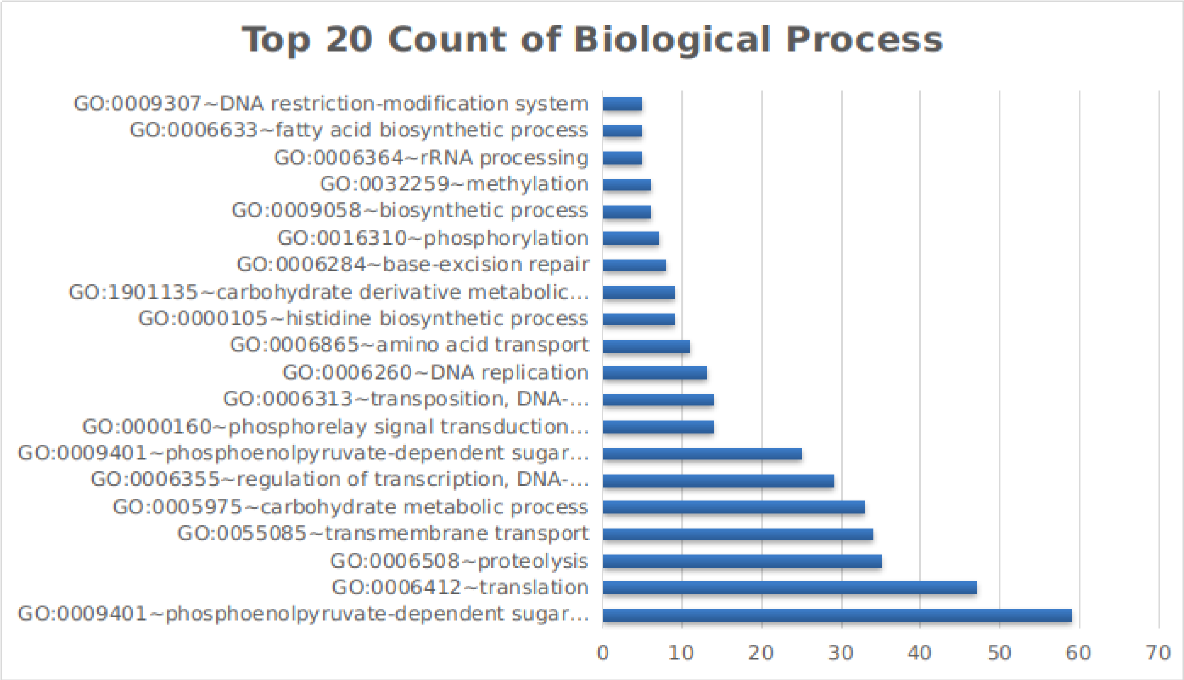

Figure S7. Top 20 Gene Ontology (GO) functions involved in biological process for *L. rhamnosus* 044AE.

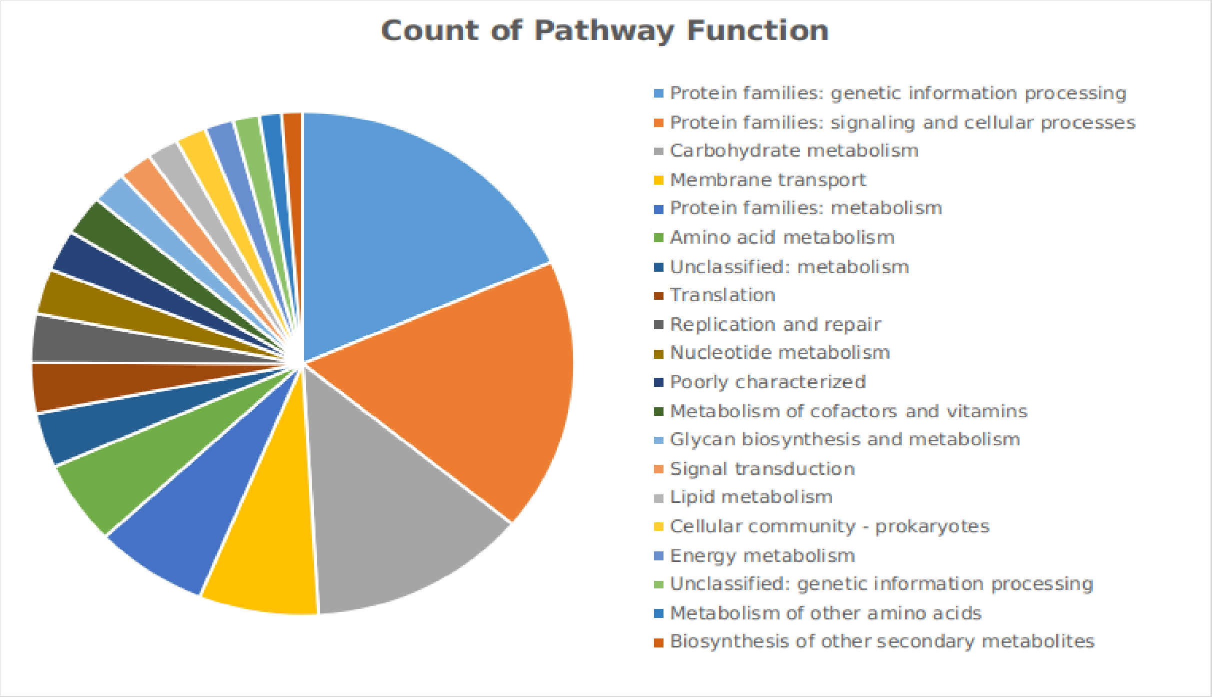

Figure S8. Pathway function associated with predicted proteins for *L. rhamnosus* 044AE.

5. Assays for *in vitro* acid, bile and temperature stability

L rhamnosus 044AE cells (2 × 10^9 CFU/mL) were exposed to bile salt solutions of 0.01, 0.1, 0.2, 0.3, 0.5, 0.7, and 1.0% at 37 °C, as well as pH 1.5, 2.5, 3.0, 5.0, and 7.0. Up to five hours, 1 millilitre of sample was taken out of each set every hour. Using the pour plate technique, viable activity was assessed.

L rhamnosus 044AE cell suspension (2 × 10^9 CFU/mL) was prepared and tested for stability at various temperatures (0, 40, 60, 80, 90 °C) for 6 h. Samples were taken every hour, immediately cooled in ice-cold water and analysed for viable activity.

Table S5. Composition of simulated digestive fluids.

Sr. No.	Electrolyte solution	Stock conc. (M)	Stock (mL) to be added to prepare 400 mL of		
			SSF	SGF	SIF
1	KCl	0.50	7.550	3.450	6.800
2	KH_2PO_4	0.50	1.850	0.450	0.800
3	$NaHCO_3$	1.00	3.400	6.250	42.500
4	NaCl	2.00	0.000	5.900	9.600
5	$MgCl_2(H_2O)_6$	0.15	0.250	0.200	1.100
6	$(NH_4)_2CO_3$	0.50	0.030	0.250	0.000
7	HCl	6.00	0.045	0.650	0.700
8	Mili-Q water		186.875	182.850	338.500

Table S6. Oral master mix.

1.	Simulated salivary fluid (SSF)	8	mL
2.	0.3 M $CaCl_2$	50	μL
3.	Water	(As per pH adjustment)	mL
4.	6 M HCl/6M NaOH	(As per pH adjustment)	μL
	Total volume	10	mL

Table S7. Gastric master mix.

1.	Simulated gastric fluid (SGF)	14.2	mL
2.	0.3 M $CaCl_2$	8.9	μL
3.	6 M HCl/6 M NaOH	(As per pH adjustment)	μL
4.	Water	(As per pH adjustment)	mL
	Total volume	16	mL

For each 8 mL of master mix, add 0.4 mL separately of pepsin stock prepared as per the Template for the harmonized *in vitro* digestion method from COST Infogest, available at: http://www.proteomics.ch/IVD/.

Table S8. Intestinal master mix.

1.	Simulated intestinal fluid (SIF)	11.95	mL
2.	0.3 M $CaCl_2$	56.28	μL
3.	Water	(As per pH adjustment)	mL
4.	Pancreatin solution	7	mL
5.	Bile solution	3.51	mL
6.	6 M HCl/6 M NaOH	(As per pH adjustment)	μL
	Total volume	28	mL

6. Cell surface properties of *L. rhamnosus* 044AE

Hydrophobicity—Microbial adhesion to hydrocarbons (MATH) was employed to quantify the hydrophobicity of *L rhamnosus* 044AE cell surface. Overnight grown culture was centrifuged, and the washed pellet was reconstituted in PBS (Phosphate Buffer Saline) at pH of 7.4. Optical density was adjusted to approximately 1.0 (A_0) at 600 nm. Equal volumes of organic solvents with varying polarities, such as xylene, ethyl acetate, and toluene, were mixed with the culture suspension, vortexed for five minutes at 1800 rpm (Labquest, Borosil, MT V012). The aqueous and organic phases were allowed to separate for 30 min at 37 °C. Optical density (A_1) at 600 nm was measured for the aqueous layer. Percentage cell surface hydrophobicity of the bacterial cells adhering to solvents was calculated using the following equation (Eq 1) [7].

$$\text{Cell Surface Hydrophobicity} = \left[1 - \frac{A_1}{A_0} \times 100\right] \quad (1)$$

Auto-aggregation—To perform the auto-aggregation test, *L rhamnosus* 044AE cells were centrifuged from an overnight culture (cultured in MRS broth at 37 °C) at 120 rpm. The pellet was washed and re-suspended in PBS to achieve an (A_0) OD_{600} of 0.3 ± 0.05. The suspension was incubated at 37 °C for 6 hours, mixed for 10 seconds, and the OD_{600} of the samples was determined (A_6) [7,8]. The auto-aggregation percentage was calculated with the following equation (Eq 2):

$$\text{Auto} - \text{aggregation } (\%) = \frac{A_0 - A_6}{A_0} \times 100 \quad (2)$$

where (A_6) represents the absorbance at 6 h, and (A_0) represents the initial absorbance.

Co-aggregation—To obtain the pellet, overnight developed cultures of pathogenic bacteria and *L rhamnosus* 044AE were centrifuged at 3500 for 15 minutes, much like autoaggregation. After two PBS washes OD_{600} was adjusted to 0.7 ± 0.05. *L rhamnosus* 044AE and pathogen cell suspensions were combined in equal volumes, and the cell suspension was incubated at 37 °C in a static environment. At 0 and 6 hours, OD_{600} was measured [8,9]. The following equation (Eq 3) was used to calculate co-aggregation (%):

$$Co - aggregation\ (\%) = \frac{\left(\frac{Apat + Aprobio}{2} - Amix\right)}{\left(\frac{Apat + Aprobio}{2}\right)} \times 100 \quad (3)$$

Apat, Aprobio = the absorbance of the pathogen and the probiotic strain at time t, Amix = the absorbance of the mixed culture at time t.

7. Mucin adhesion assay

Overnight of *L rhamnosus* 044AE were prepared in 2 stages to obtain an OD_{600} of approximately 1.5 (Pellets twice washed and reconstituted with sterile PBS). 120 µL of mucin agar (pH 6.8) was placed in each of the 96 well plates (Nunc® Edge 2.0, Sigma) together with 100 µL of the suspensions (OD_{600} ~1.5). The agar control was a suspension cultured of 1% (w/v) bacteriological agar. The plates were incubated at 50 rpm at 37 °C. Following a 90-minute incubation period, the liquid phase was removed and the wells were washed twice with 100 µL of PBS to remove any loosely adhering cells. Using a sterile spatula, solidified mucin and bacteriological agar were removed and homogenized in 5 millilitres of peptone saline. The material was tested for cell viability by pour plate [10]. Viable activities were determined in terms of CFU/well for both agar control and mucin test well.

8. Adhesion to Caco-2 cell lines

Caco-2 cells were cultured in MEM media with 20% fetal bovine serum at 37 °C and 5% CO_2. Media was refreshed every 2–3 days. Caco-2 cells (1 × 10^5 cells/mL) were seeded in six-well plates and incubated at 37 °C and 5% CO_2. Medium was replaced every 48 hours until cells reached 80% confluency. Spent medium was replaced with MEM (antibiotic-free) and incubated at 37 °C for 30 min. Cells were washed twice with PBS (pH 7.4). 1 mL MEM (serum and antibiotic-free) was added and incubation was done at 37 °C for 30 min. Bacterial isolates (1 × 10^9 CFU in 1 mL MEM) were added to wells and plates were incubated at 37 °C, 5% CO_2 for 2 hours. Monolayer was washed five times with PBS to remove non-adherent bacteria. Cells were fixed with 2 mL methanol for 10 min and stained with 3 mL Giemsa (1:20 in PBS) for 20 min. After rinsing with distilled water, monolayer was air dried and examined under a 40X microscope. Bacteria were counted in 20 random fields and adhesion was classified as non-adhesive (≤40), adhesive (41–100), or strongly adhesive (>100).

Fir the calculation of percent adhesion, monolayer was washed five times with PBS to remove non-adherent bacteria. Cells were deattached using 1 mL 0.25% trypsin–EDT and incubated for 15 min.

Cell-bacteria suspension was serially diluted in saline and plated on MRS agar. Viable bacteria were enumerated after incubation. Adhesion was calculated as follows (Eq 4).

$$\text{Percent adhesion} = \frac{B1}{B0} \times 100 \quad (4)$$

where B0 and B1 are initial and final CFU counts.

Table S9. Gene prediction statistics.

Summary	Number
Genes (total)	2870
CDS (total)	2754
tmRNAs	1
tRNAs	61
miscRNA	39
rRNA	15

Table S10. List of some of the important AMR and virulence genes which were absent in the genome of *L. rhamnosus* 044AE.

Important AMR genes				
Accession	AMR gene family	Resistance Mechanism	Identity	Remark
ARO:3003438	elfamycin resistant EF-Tu	antibiotic target alteration	73.49	Absent
ARO:3003078	daptomycin resistant liaR	antibiotic efflux;antibiotic target alteration	75.51	Absent
ARO:3003735	antibiotic resistant fusA	antibiotic target alteration	71.42	Absent
ARO:3000024	ATP-binding cassette (ABC) antibiotic efflux pump	antibiotic efflux	52.83	Absent
ARO:3003778	antibiotic resistant ndh	antibiotic target alteration	29.279	Absent
Important virulence genes				
VFDB ID	Gene	Product	Identity	Remark
VFG041185 (gi:269139781)	evpH	type VI secretion system protein EvpH [EVP (E. tarda virulent protein) (SS189)] [*Edwardsiella tarda* EIB202]	38.61	Absent
VFG006826 (gi:16803417)	lisR	two-component response regulator [LisR/LisK (CVF253)] [*Listeria monocytogenes* EGD-e]	30.18	Absent
VFG002165 (gb|NP_815739)	efaA	endocarditis specific antigen [EfaA (VF0354)] [*Enterococcus faecalis* V583]	29.41	Absent
VFG019048 (gi:76787756)	psaA	manganese ABC transporter, manganese-binding adhesion liprotein [Pneumococcal surface antigen A/Metal binding protein SloC (CVF181)] [*Streptococcus pneumoniae* CGSP14]	52.22	Absent
VFG044147(gi:16766086)	iroC	ABC transporter protein [Salmochelin (IA021)] [*Salmonella enterica subsp. enterica serovar Typhimurium* str. LT2]	26.23	Absent

Table S11. Cell surface properties shown by *Lactobacillus rhamnosus* 044AE.

Cell surface Properties		
	Adhesion to	% Adhesion
Adhesion to non-polar solvent	Xylene	1.5 ± 0.47
	Toluene	7.83 ± 1.01
	Chloroform	13.03 ± 1.16
	Ethyl acetate	24.86 ± 3.12
Autoaggregation	044AE	7.34 ± 0.94
Co aggregation	044AE + *C. perfringens*	45.4 ± 1.18
	044AE + *S. enterica*	16.38 ± 9.29

Table S12. Antioxidant activity of *Lactobacillus rhamnosus* 044AE.

Sample	% Radical scavenging activity
100 µg/mL std. Ascorbic acid	39.45
044AE	16.82 ± 4.64

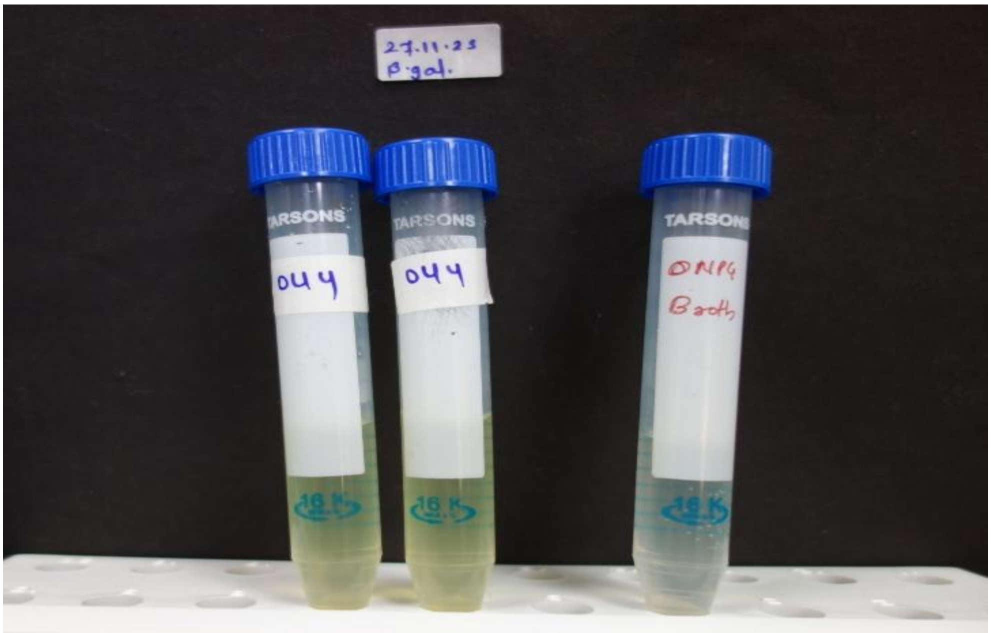

Figure S9. Yellow coloration of ONPG broth due to production of β-galactosidase by *L rhamnosus* 044AE.

(A)

(B)

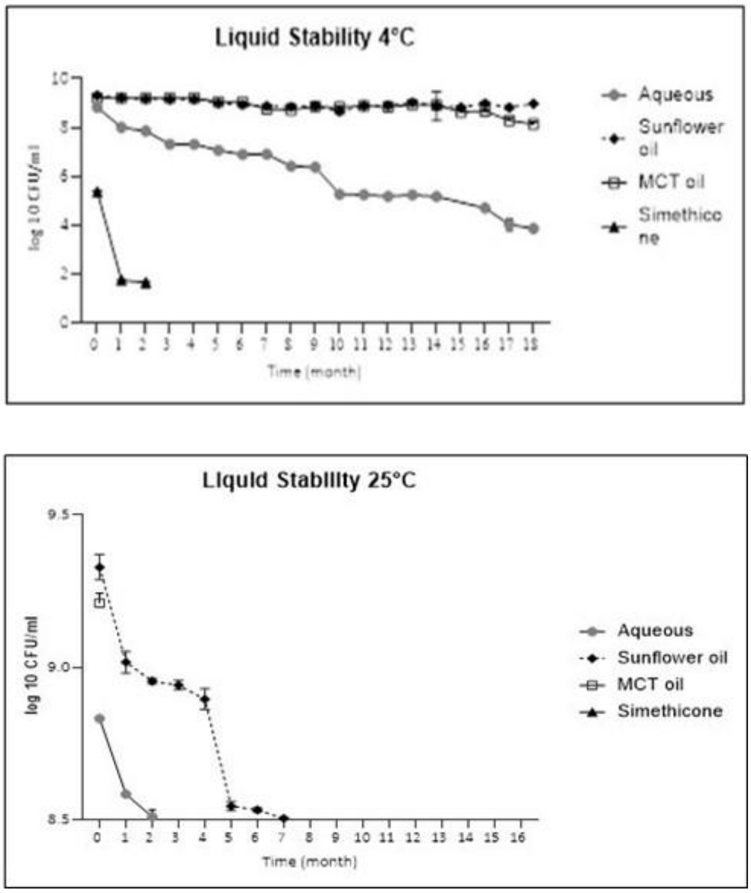

Figure S10. Viability of *L rhamnosus* 044AE cells in different matrices for 1, 2, 3, 6 and 9 months at 4°C (A) and 25°C (B).

AIMS Press

© 2026 the Author(s), licensee AIMS Press. This is an open access article distributed under the terms of the Creative Commons Attribution License (https://creativecommons.org/licenses/by/4.0)
